# Supplementary material for: Changes in Local and Systemic Adverse Effects following Primary and Booster Immunisation against COVID-19 in an Observational Cohort of Dutch Healthcare Workers Vaccinated with BNT162b2 (Comirnaty®)
Source: Vaccines (Basel). 2023 Dec 29;12(1):39. doi: 10.3390/vaccines12010039 (PMC10821042; doi:10.3390/vaccines12010039)
Supplement: Supplementary file 1 [file vaccines-12-00039-s001.zip › vaccines-2800578-supplementary.pdf]

English translation first; Dutch original below.

<u>QUESTIONNAIRE 1: TO BE FILLED IN AFTER THE FIRST VACCINE</u>

1.a Have you ever had a <u>proven</u> COVID-19 infection?

□ YES □ NO, continue to question 2 (next page)

1.b In what way has infection been proven?

□ Positive PCR	□ Positive SEROLOGY (antibodies)	□ Test type unknown
On what date? ____/_____/20___	On what date? ____/_____/20___	On what date? ____/_____/20___

1.c Have you experienced symptoms during this infection?

□ YES □ NO, continue to question 2 (next page)

1.d How severe do you feel your symptoms were?

□ MILD	□ MEDIUM	□ SEVERE
Continue to question 2	Continue to question 2	Continue to question 1e

1.e How long did these severe symptoms persist?

□ ONE DAY □ SEVERAL DAYS □ ONE WEEK □ SEVERAL WEEKS

2a. Did you experience any of the following adverse effects following your first vaccination dose?

If not, please tick "no"

If you did, please indicate how severe you feel your symptoms were (MILD, MEDIUM, SEVERE), and indicate at how many days after vaccination these effects began.

**Adverse effects**	**Answer**				**After … days:**
Local injection site pain	□ NEE	□	□ MATIG	□ ERNSTIG	1 / 2 / 3 / 4 / 5
Fatigue	□ NEE	□	□ MATIG	□ ERNSTIG	1 / 2 / 3 / 4 / 5
Headache	□ NEE	□	□ MATIG	□ ERNSTIG	1 / 2 / 3 / 4 / 5
Myalgia / muscle ache	□ NEE	□	□ MATIG	□ ERNSTIG	1 / 2 / 3 / 4 / 5
Chills	□ NEE	□	□ MATIG	□ ERNSTIG	1 / 2 / 3 / 4 / 5
Arthralgia / joint pains	□ NEE	□	□ MATIG	□ ERNSTIG	1 / 2 / 3 / 4 / 5
Fever	□ NEE	□	□ MATIG	□ ERNSTIG	1 / 2 / 3 / 4 / 5
Allergic reaction / rash	□ NEE	□	□ MATIG	□ ERNSTIG	1 / 2 / 3 / 4 / 5
Severe allergic reaction	□ NEE	□ JA			1 / 2 / 3 / 4 / 5

2b. Did you call in sick because of these effects?

□ YES □ NO

3. Please indicate if you have any further comments

____

ATTENTION:

The following questionnaire should be filled out <u>one</u> week following the second dose.

1.a Have you ever had a <u>proven</u> COVID-19 infection?

□ YES   □ NO, continue to question 2 (next page)

1.b In what way has infection been proven?

□ Positive PCR<br><br>On what date?<br>____/_____/20___	□ Positive SEROLOGY (antibodies)<br><br>On what date?<br>____/_____/20___	□ Test type unknown<br><br>On what date?<br>____/_____/20___

1.c Have you experienced symptoms during this infection?

□ YES   □ NO, continue to question 2 (next page)

1.d How severe do you feel your symptoms were?

□ MILD	□ MEDIUM	□ SEVERE
Continue to question 2	Continue to question 2	Continue to question 1e

1.e How long did these severe symptoms persist?

□ ONE DAY   □ SEVERAL DAYS   □ ONE WEEK   □ SEVERAL WEEKS

2a. Did you experience any of the following adverse effects following your second vaccination dose?
If not, please tick “no”
If you did, please indicate how severe you feel your symptoms were (MILD, MEDIUM, SEVERE), and indicate at how many days after vaccination these effects began.

Adverse effects	Answer				After … days:
Local injection site pain	□ NEE	□	□ MATIG	□ ERNSTIG	1 / 2 / 3 / 4 / 5
Fatigue	□ NEE	□	□ MATIG	□ ERNSTIG	1 / 2 / 3 / 4 / 5
Headache	□ NEE	□	□ MATIG	□ ERNSTIG	1 / 2 / 3 / 4 / 5
Myalgia / muscle ache	□ NEE	□	□ MATIG	□ ERNSTIG	1 / 2 / 3 / 4 / 5
Chills	□ NEE	□	□ MATIG	□ ERNSTIG	1 / 2 / 3 / 4 / 5
Arthralgia / joint pains	□ NEE	□	□ MATIG	□ ERNSTIG	1 / 2 / 3 / 4 / 5
Fever	□ NEE	□	□ MATIG	□ ERNSTIG	1 / 2 / 3 / 4 / 5
Allergic reaction / rash	□ NEE	□	□ MATIG	□ ERNSTIG	1 / 2 / 3 / 4 / 5
Severe allergic reaction	□ NEE	□ JA			1 / 2 / 3 / 4 / 5

2b. Did you call in sick because of these effects?

□ YES □ NO

3. Please indicate if you have any further comments

____

VRAGENLIJST 1: IN TE VULLEN NA DE 1E VACCINATIE

1.a Heeft u een bewezen COVID-19 infectie gehad?

□ JA   □ NEE, ga verder naar vraag 2 (volgende pagina)

1.b Op welke manier is de infectie bewezen?

□ Positieve PCR	□ Positieve SEROLOGIE (antistoffen)	□ TEST-TYPE ONBEKEND
Op welke datum? ____/_____/20___	Op welke datum? ____/_____/20___	Op welke datum? ____/_____/20___

1.c Heeft u klachten gehad tijdens deze infectie?

□ JA   □ NEE, ga verder naar vraag 2 (volgende pagina)

1.d Hoe ernstig heeft u deze klachten ervaren?

□ LICHT	□ MATIG	□ ERNSTIG
ga verder naar vraag 2	ga verder naar vraag 2	ga verder naar vraag 1e

1.e Hoe lang hielden deze ernstige klachten aan?

□ EEN DAG   □ ENKELE DAGEN   □ EEN WEEK   □ ENKELE WEKEN

2a. Heb je na het krijgen van je eerste vaccinatie last gehad van de volgende bijwerkingen?

Indien nee, kruis dan "NEE" aan.

Indien ja, geef dan aan hoe ernstig je deze bijwerking ervaren hebt (LICHT/MATIG/ERNSTIG) en omcirkel na hoeveel dagen deze klacht begon (dag 0 t/m 5).

**Bijwerkingen**	**Antwoord**				**Na hoeveel**
Matige pijn op de injectieplaats	□ NEE	□	□ MATIG	□ ERNSTIG	1 / 2 / 3 / 4 / 5
Vermoeidheid	□ NEE	□	□ MATIG	□ ERNSTIG	1 / 2 / 3 / 4 / 5
Hoofdpijn	□ NEE	□	□ MATIG	□ ERNSTIG	1 / 2 / 3 / 4 / 5
Spierpijn	□ NEE	□	□ MATIG	□ ERNSTIG	1 / 2 / 3 / 4 / 5
Rillingen	□ NEE	□	□ MATIG	□ ERNSTIG	1 / 2 / 3 / 4 / 5
Gewrichtspijn	□ NEE	□	□ MATIG	□ ERNSTIG	1 / 2 / 3 / 4 / 5
Koorts	□ NEE	□	□ MATIG	□ ERNSTIG	1 / 2 / 3 / 4 / 5
Allergische reactie:	□ NEE	□	□ MATIG	□ ERNSTIG	1 / 2 / 3 / 4 / 5
Ernstige allergische reactie	□ NEE	□ JA			1 / 2 / 3 / 4 / 5

2b. Heb je je ziek moeten melden i.v.m. deze klachten?

□ JA □ NEE

3. Nog bijzonderheden? Deze graag kort noteren s.v.p.

____

LET OP:

De volgende vragenlijst dient pas een week ná de twee vaccinatie ingevuld te worden

VRAGENLIJST 2: IN TE VULLEN EEN WEEK NA DE 2[E] VACCINATIE

1.a Heeft u een bewezen COVID-19 infectie gehad?

◻ JA ◻ NEE, ga verder naar vraag 2 (volgende pagina)

1.b Op welke manier is de infectie bewezen?

◻ Positieve PCR	◻ Positieve SEROLOGIE (antistoffen)	◻ TEST-TYPE ONBEKEND
Op welke datum? ____/_____/20___	Op welke datum? ____/_____/20___	Op welke datum? ____/_____/20___

1.c Heeft u klachten gehad tijdens deze infectie?

◻ JA ◻ NEE, ga verder naar vraag 2 (volgende pagina)

1.d Hoe ernstig heeft u deze klachten ervaren?

◻ LICHT	◻ MATIG	◻ ERNSTIG
ga verder naar vraag 2	ga verder naar vraag 2	ga verder naar vraag 1e

1.e Hoe lang hielden deze ernstige klachten aan?

◻ EEN DAG ◻ ENKELE DAGEN ◻ EEN WEEK ◻ ENKELE WEKEN

2a. Heb je na het krijgen van je tweede vaccinatie last gehad van de volgende bijwerkingen?

Indien nee, kruis dan "NEE" aan.

Indien ja, geef dan aan hoe ernstig je deze bijwerking ervaren hebt (LICHT/MATIG/ERNSTIG) en omcirkel na hoeveel dagen deze klacht begon (dag 0 t/m 5).

**Bijwerkingen**	**Antwoord**				**Na hoeveel**
Matige pijn op de injectieplaats	□ NEE	□	□ MATIG	□ ERNSTIG	1 / 2 / 3 / 4 / 5
Vermoeidheid	□ NEE	□	□ MATIG	□ ERNSTIG	1 / 2 / 3 / 4 / 5
Hoofdpijn	□ NEE	□	□ MATIG	□ ERNSTIG	1 / 2 / 3 / 4 / 5
Spierpijn	□ NEE	□	□ MATIG	□ ERNSTIG	1 / 2 / 3 / 4 / 5
Rillingen	□ NEE	□	□ MATIG	□ ERNSTIG	1 / 2 / 3 / 4 / 5
Gewrichtspijn	□ NEE	□	□ MATIG	□ ERNSTIG	1 / 2 / 3 / 4 / 5
Koorts	□ NEE	□	□ MATIG	□ ERNSTIG	1 / 2 / 3 / 4 / 5
Allergische reactie:	□ NEE	□	□ MATIG	□ ERNSTIG	1 / 2 / 3 / 4 / 5
Ernstige allergische reactie	□ NEE	□ JA			1 / 2 / 3 / 4 / 5

2b. Heb je je ziek moeten melden i.v.m. deze klachten?

□ JA □ NEE

3. Nog bijzonderheden? Deze graag kort noteren s.v.p.

____
